# Psychiatrists' follow-up of identified metabolic risk: a mixed-method analysis of outcomes and influences on practice

**DOI:** 10.1192/pb.bp.114.049379

**Published:** 2016-10

**Authors:** Sue Patterson, Kathleen Freshwater, Nicole Goulter, Julie Ewing, Boyd Leamon, Anand Choudhary, Vikas Moudgil, Brett Emmerson

**Affiliations:** 1 Metro North Mental Health, Queensland, Australia; 2 Griffith University, Queensland, Australia; 3 Queensland University of Technology, Queensland, Australia; 4 The University of Queensland, Australia

## Abstract

**Aims and method** To describe and explain psychiatrists' responses to metabolic abnormalities identified during screening. We carried out an audit of clinical records to assess rates of monitoring and follow-up practice. Semi-structured interviews with 36 psychiatrists followed by descriptive and thematic analyses were conducted.

**Results** Metabolic abnormalities were identified in 76% of eligible patients screened. Follow-up, recorded for 59%, was variable but more likely with four or more abnormalities. Psychiatrists endorse guidelines but ambivalence about responsibility, professional norms, resource constraints and skills deficits as well as patient factors influences practice. Therapeutic optimism and desire to be a ‘good doctor’ supported comprehensive follow-up.

**Clinical implications** Psychiatrists are willing to attend to physical healthcare, and obstacles to recommended practice are surmountable. Psychiatrists seek consensus among stakeholders about responsibilities and a systemic approach addressing the social determinants of health inequities. Understanding patients' expectations is critical to promoting best practice.

The metabolic syndrome comprises a cluster of five metabolic abnormalities directly linked to development of cardiovascular disease,^1^ a leading cause of premature mortality among people diagnosed with schizophrenia.^2^ Diagnosis of metabolic syndrome, according to the International Diabetes Federation,^3^ is appropriate when central obesity co-occurs with any two other specified abnormalities: raised triglycerides, plasma glucose or blood pressure, and reduced high-density lipoprotein cholesterol. Not least because treatment with antipsychotic medication contributes to metabolic dysfunction, clinical guidelines have long recommended that psychiatrists attend to the metabolic health of patients, screening and intervening to reduce risk.^4^ Practice, however, is highly variable;^5,6^ metabolic abnormalities go underdiagnosed and often fail to elicit appropriate response.^7–9^ Hence, various interventions have sought to improve practice. Some – for example, formalising protocols^10^ and scheduling screening at service, rather than individual level^9^ – have increased monitoring. However, improvement in health outcomes requires intervention; when abnormalities are detected, further investigations, patient education and/or pharmacological or lifestyle interventions targeting weight reduction and increased exercise are recommended as minimally adequate responses.^4^ Because guideline implementation is complex, and shaped by interacting guideline, system, professional and patient factors, arguments are made that interventions to support implementation be multifaceted and contextually relevant.^11,12^ A detailed understanding of how psychiatrists currently respond to metabolic dysfunction in people prescribed antipsychotics and what influences practice is critical to intervention design, and will augment the ‘sparse and inconclusive’ knowledge^12^ about embedding evidence-based practice in mental healthcare.

Against this background we set out to describe the rate and nature of follow-up of metabolic abnormalities identified during routine screening, and influences on psychiatrists' follow-up practice.

## Method

A mixed-method observational study,^13^ combining audit of clinical records with qualitative investigation of influences on practice, was conducted.

### Setting

Two of three sites of an Australian mental health service (AMHS) were chosen for the study. The service encompasses inner city, suburban and regional areas with a sociodemographically diverse population of about 1 million, and are community based. Multidisciplinary teams, linked to in-patient units through team psychiatrists, provide assessment and treatment across geographically defined areas. Care is coordinated by designated (allied health or nursing) clinicians and patients prescribed antipsychotic medication consult with psychiatrists or training psychiatrists each month in out-patient clinics. At the time of the study, service policy obliged psychiatrists to complete metabolic screening for patients prescribed clozapine and olanzapine in designated months and promote adherence to the clinical algorithm developed by Jackie Curtis and colleagues.^14^ The algorithm, endorsed by the Royal College of Psychiatrists as The Lester Protocol,^15^ is regularly reviewed at clinical meetings and copies are posted in consulting rooms.

### Data collection and analysis

Data were collected and analysed by authors, who are all employed by the AMHS.

**Fig. 1:**
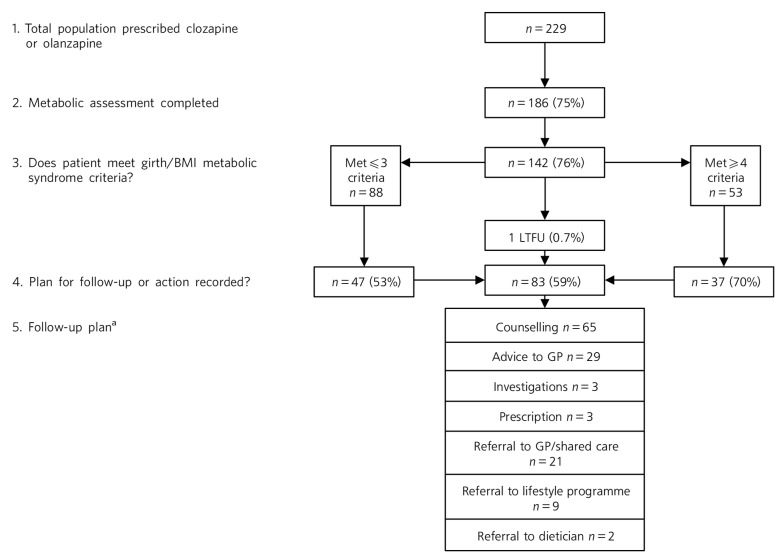
Assessment of eligible patients and follow-up of those meeting International Diabetes Federation body mass index (BMI)/girth criteria for metabolic syndrome. GP, general practitioner; LTFU, lost to follow-up. a. Multiple responses were permitted.

The audit sample was selected as shown in Fig. 1. We reviewed monitoring data (routinely collated by the service) for November 2013 at one site, identifying patients whose central obesity (body mass index (BMI)⩾30 or girth circumference ⩾80 cm for females and 94 cm for males) indicated risk for metabolic syndrome.^3^ This criterion was selected because it is fundamental to diagnosis of metabolic syndrome and the algorithm recommends clinical response. Extracted from charts for each patient were demographics, treating doctor and notation regarding follow-up for metabolic abnormalities in the month after screening. Follow-up actions were categorised based on Wilson and colleagues^9^ as ‘counselling’ (i.e. notes stated ‘counselled’ or indicated conversation with the patient about screening results, lifestyle change or interventions), ‘advice to general practitioner’ (GP), ‘investigations’, ‘prescription of hypoglycaemic or hypolipidaemic agents’, ‘referral to GP, lifestyle programme or dietician’ and ‘other’. ‘Other’ follow-up actions were then categorised. Where multiple actions were recorded, each was counted separately. Data were entered into a database for descriptive analysis. Comparisons were made using χ^2^ and *t*-tests dependent on data type, with *P*<0.05 regarded as statistically significant.

Qualitative data were collected in semi-structured individual interviews (*n* = 9) and focus groups (*n* = 3 with 12, 9 and 7 participants) between February and June 2014. Psychiatrists invited to interview were site clinical directors and psychiatrists known through audit to routinely or seldom follow up abnormalities. Psychiatrists were recruited to focus groups when attending routine educational meetings given over to the study at each site. Informed consent was obtained prior to data collection.

In interviews and focus groups, audit findings were first summarised before participants were prompted to explore three questions:

1. What is/are your responsibility/ies in management of physical health of patients?
2. How do you action these responsibilities?
3. What influences your responses to identified abnormalities?

Data were recorded and transcribed immediately following interviews. To enable exploration of expressed views, data collection and analysis using the framework approach^16^ were concurrent. After each transcript was generated, data were allocated to a cell in an initial frame, representing source and research question with multiple allocations possible. Next, a process of constant comparison was used to discern patterns and exceptions in data within cells and between data sources. While maintaining links to source, related data were grouped and descriptively labelled, and subthemes were identified. Credibility of the analysis was promoted by: checking of developing findings in ongoing interviews by members; critical dialogue between authors with psychiatrist members of the team, drawing on clinical experiences to check resonance; review of findings by an academic psychiatrist acting as a critical friend; and a service committee that oversees metabolic monitoring.

Certified Australian Human Research Ethics Committees approved the qualitative study (HREC/13/QPCH/200), and granted exemption from review for the audit (HRECEC00172).

## Results

Metabolic monitoring was completed for 186 of 229 eligible patients (75%) and three-quarters (*n* = 142; 76%), including 95 men, met central obesity criteria. Patients were aged 23–73 years (mean 45). Men were significantly younger than women (mean 42.6, s.d. = 0.2 years *v.* mean 49.3, s.d. = 10.9 years; *t*(140) = 3.8, *P*<0.01). Central obesity was the only metabolic syndrome criterion met for a small minority of patients (*n* = 19; 13%). Fifty-three (36%) patients (half of whom were men) met four or more criteria. Records indicated pre-screen diagnoses of diabetes mellitus in 11 patients, and prescription of hyperglycaemic and/or hypolipidaemic medication in 17 patients. No new diagnoses of metabolic syndrome or diabetes mellitus were recorded.

Notation regarding follow-up of abnormalities was found in a majority (*n* = 83; 59%) of the 141 patients in contact with the service following screening. Although neither BMI nor gender were related to follow-up, patients with four or more detected abnormalities were significantly more likely than those who had fewer than four abnormalities detected to have action recorded in charts (*n* = 37, 70% *v. n* = 47, 53%; χ^2^(4,141) = 145.72, *P*<0.01).

The ‘quality’of records and type of follow-up actions were variable, with individual doctors consistently using a similar approach/notation for each patient. About a third routinely recorded follow-up involving multiple actions (e.g. counselling, dietary advice, referral to lifestyle programmes and/or GP). More commonly, a single action, typically some form of counselling, was recorded. Follow-up coded as ‘counselling’ was diverse; a minority of doctors detailed the approach (e.g. motivational interviewing) and recommendations; most entries stated ‘counselled’ or ‘psychoed’ (indicating some form of psychoeducation). ‘Other’ follow-up actions (*n* = 15) included letters reporting results to health/Social Service providers, change of antipsychotic medication (*n* = 1), and specified a patient activity (e.g. food diary; *n* = 3). Infrequently, notes indicated that patients refused referrals (*n* = 3) or were already engaged in programmes or attempting to self-mange weight. Patients living in the service community rehabilitation facility were referred to the in-house healthy living programme (*n* = 7).

### Influences on follow-up actions

Thirty-six psychiatrists (including 23 trainees; 85% of those working across the services) and two registered medical officers participated in interviews of around 15 min duration and focus groups lasting 30–55 min. Participants were heterogeneous with respect to age, experience, clinical specialty and training locality, but several had completed their medical degrees overseas. The sample included doctors known through audit findings and by self-description to ‘consistently’, ‘sometimes’ or ‘seldom’ instigate follow-up of metabolic abnormalities.

Analysis identified diverse influences on practice in six themes. In presenting the themes we describe the prevalence of an opinion/point of view by attributing it to a non-specific number of participants using the terms ‘most/majority’ (group consensus or multiple instances), ‘some’ (a view expressed by most of those interviewed but not widely endorsed), and ‘few/minority’ (a divergent opinion or one expressed only occasionally). Illustrative quotations are attributed to an interview participant or a focus group (FG).

#### 1. Knowledge and endorsement of guidelines regarding metabolic management

All participants knew about, and most endorsed, guidelines acknowledging responsibility for attending to side-effects of prescribed medication. However, concern was expressed about increasing demands on psychiatrists managing acutely unwell patients within an underresourced health system. A strongly expressed minority opinion was that specialist psychiatry roles were being diluted as requirements to undertake ‘generalist’ tasks increased.

Psychiatrists were also aware that performance was being monitored by the service: ‘it is certainly in our consciousness’, said one consultant. Mixed views were expressed about the impact of this, with administrative targets and ‘authoritarian’ surveillance of practice described as devaluing clinical judgement, potentially leading to superficial change – ‘doing it because we should’ (FG2, consultant), rather than meaningful engagement with patients and enduring practice shifts.

#### 2. Don't rock the boat!

Informal normative pressure, a key influence on practice, played out in various ways.

‘Psychiatrists turn their backs toward physical illnesses because it's handed on generation to generation … tradition to follow the seniors’ (interview 2, registrar).

Psychiatrists in training commonly reported modelling practice on that of whichever consultant was providing supervision or work unit norms. Although a minority welcomed opportunities to challenge the perceived status quo, most trainees considered it inappropriate to ‘rock the boat’, particularly when involvement was time limited.

‘Clients [at rehabilitation unit] all smoke and they're obese … I'm just here for three months so I shouldn't change anything’ (interview 3, trainee).

Concerns related to being perceived unfavourably by colleagues as ‘a new broom’ and disruption of continuity of care for patients.

#### 3. Shared responsibility

Describing health as complex (involving genetics, lifestyle and social factors), psychiatrists were concerned about ‘blaming individuals’ and/or antipsychotic medication for metabolic dysfunction. The consensus was that a systemic response (encompassing social programmes and multiple agencies) was required to improve outcomes of people with severe mental illness (SMI). That psychiatry and mental health services had a role to play was agreed, but opinions diverged around what that role should be. Consistent with ‘referral’ being the most common follow-up, psychiatrists typically considered (non-medical) mental health clinicians and GPs as best placed to manage physical health. They argued that their own potential as a ‘bit player’ with multiple competing demands to influence outcomes should not be overstated.

‘You can't be a one stop shop’ (interview 4, trainee).

#### 4. Patient characteristics and expectations

‘[Psychiatrists] have to be realistic … patients come from lower socioeconomic backgrounds and unfortunately, this is indicative of poor nutritional choices, poor exercise regimes’ (FG2, trainee)

Noting that improvement in health was dependent on behavioural change, psychiatrists described patient characteristics (i.e. age, culture, social class and living circumstances) as key influences on practice. They spoke of informally assessing readiness for change and likelihood of follow-up recommendations being enacted.

‘If you have someone who is fat and unmotivated, there's no point – you can advise all you like and nothing is going to change’ (interview 7, consultant, high follow-up rate).

Patients were described as typically lacking interest in physical health and ‘pre-contemplative’, with low motivation for lifestyle change attributed variously to symptoms of mental illness (e.g. ‘concerned with what's going on in their heads’), viewing a large body positively (e.g. ‘they think they are like body builders and they do not want to be skinny’), and repeated unsuccessful attempts at changing behaviour/losing weight.

‘In the denial phase … so once we convince them that it is unhealthy, then we might get them on a different diet’ (interview 4, trainee).

Although typically hoping for positive outcomes, several questioned whether behaviour change was possible and whether diet/exercise could work with this (or any) population. Psychiatrists reported discontinuing recommending interventions when this seemed futile, when referrals and/or discussion of physical health were declined. Moreover, some patients were described as considering physical health out of scope: ‘not what [patients] come to us for’.

#### 5. Resources and capacity

Environmental resources (primarily their lack) and utilisation featured in psychiatrists' explanations of metabolic monitoring and follow-up. References to ‘time’ were recurrent but divergent views were expressed. Most psychiatrists identified time as the key barrier to follow-up, noting that consultations were barely long enough to complete and document essential psychiatric assessments. With metabolic dysfunction, especially obesity, considered a chronic rather than acute problem, psychiatrists' identified legislative requirements and risk/safety as their primary concern.

‘You see a patient with alcohol problems, smoking, obese, you suggest “You should stop smoking, stop drinking alcohol … but sorry, I don't have time today” (…) you have to concentrate on mental health’ (FG3, trainee).

Others, although acknowledging time constraints, proposed that how consultations were conducted mattered. Those who routinely undertook follow-up identified two enabling practices: routine discussion of well-being and physical health, and working after hours to complete clinical notes and referral letters, because they derived professional satisfaction from being ‘good doctors’.

‘You can't always be talking about the voices, these people are not acutely unwell and their recovery is about more than symptoms’ (interview 6, consultant, high follow-up rate).

A minority of psychiatrists said that pressure to reduce service costs influenced their decisions, particularly about ordering further tests, as one said.

‘Yes, but who pays? We get mixed messages in the current political climate … promoted highly that we give the highest quality healthcare but the reality is that it's a minimalist budget; the cost is to the consumer’ (FG1, trainee).

Awareness, endorsement and accessibility of interventions influenced practice. Several psychiatrists reported not knowing what was available or how to refer, and others questioned the evidence supporting intervention with ‘this population’.

#### 6. Motivation and capability

Self-perceived capability and views about ‘appropriate’ practice also surfaced as influential. Motivation and willingness to engage patients in discussion of lifestyle and particularly weight varied widely. A minority of psychiatrists attributed their own reluctance to discuss physical health (especially weight) to thinking this inappropriate or not knowing the right words, or expressed concern that mentioning of weight or lifestyle could cause offence and disrupt therapeutic alliance with the patient. Most psychiatrists spoke of adapting their approach to fit each patient's perceived openness to discussion and likelihood of change but one advocated a frank approach:

‘I, quite brutally might say “If you do not change your lifestyle you are going to die 20 years younger”, I put it on the line … some of them might accept that but for some it's quite a shock and others just don't care’ (FG2, trainee).

The use (or not) of medication for metabolic dysfunction/obesity was robustly debated. With few reporting routinely prescribing lipid-lowering agents, psychiatrists commonly regarded themselves as ‘consciously incompetent’ to do so (interview 8, consultant). Psychiatrists spoke of uncertainty about dosing and drug interactions, particularly if adherence was problematic, and inability to monitor response if a patient's service contact was irregular. Many observed that maintaining currency with psychiatric evidence constrained their ability to engage with ‘other’ literature, and reported lacking opportunities to develop expertise.

‘As you become more senior in psychiatry, basic medical skills like interpreting test results are lost or diminish’ (FG3, consultant).

The view that prescribing non-psychotropic medications was beyond scope had been reinforced when physicians/endocrinologists from whom advice had been sought in various circumstances were unable to articulate precise recommendations. Additionally, and specifically in relation to metformin, psychiatrists questioned the ethics of prescribing off-label.

‘We could prescribe for insulin resistance but really we're prescribing for obesity. I'm not sure that's ethical’ (interview 7, consultant).

Whereas some participants considered clinical algorithms (such as that proposed by Curtis *et al*^14^) helpful, others drew attention to difficulty translating a general guideline (e.g. ‘think about metformin’) into practice with an individual.

‘If it's not core business, then a clear treatment algorithm is required – at the moment there are no clear guidelines, which means I'm not confident prescribing’ (discussion 2, trainee).

### Suggestions for practice improvement

Psychiatrists identified various solutions to perceived problems. Describing unrelenting pressure to mitigate risk and engage patients who saw no need for psychiatric treatment (never mind physical healthcare), some proposed establishment of clinics to be run by junior doctors or specialist metabolic monitoring roles. Others focused on process, particularly coordination and communication with GPs. Simple referral processes and efficient feedback mechanisms were considered essential if patients were to avoid ‘falling through systemic gaps’. More fundamentally, psychiatrists called for consensus among stakeholders within (psychiatrists, mental health clinicians and administrators) and external to (GPs and non-government agencies) mental health services about responsibilities – knowing who does what and when. However, psychiatrists noted that none of these strategies could improve outcomes unless patients engaged.

## Discussion

This study revealed, and explained from the psychiatrists' perspective, inconsistency in response to metabolic abnormalities which were identified during routine screening of patients who were prescribed antipsychotic medication. Most patients with central obesity were followed up, and the consistently comprehensive response of some doctors indicates that observed deficits and delays in guideline implementation are remediable. However, such positive results were achieved in challenging circumstances with multiple barriers to best practice identified. The time constraints, focus on psychiatric symptoms, concerns about role diffusion, and beliefs that patients are unable/unwilling to modify lifestyles have all been described as constraining attention to physical health.^7,17–22^ This study, however, extends these findings by contextualising such proximal influences on practice. In combination with the multiple demands on their limited time, psychiatrists' understanding of the health of people with SMI as a product of dynamic interactions between personal characteristics and social conditions beyond their control contributes to ambivalence about their roles and observed clinical inertia.^23^ Hence, rather than seeing themselves as singularly responsible, they construe themselves as contributing to what is necessarily a systemic solution to the excess morbidity among people with SMI.

### Study limitations

Implications should be considered in light of limitations, which constrain generalisability. Findings were generated within particular, sociopolitically and geographically situated services. The psychiatrists who took part were not randomly selected and partly because the services have been encouraging attention to physical health of patients for nearly 3 years, their views and experiences may differ from peers working elsewhere. Although the depth and breadth of discussion indicates that psychiatrists felt able to share views openly, data were necessarily shaped by the setting and process of collection and researcher characteristics. In mitigation, we note that the sample was substantial and diverse in relation to level and location of training and duration of practice within AMHS, and member-checking demonstrated credibility of findings.

### Implications for practice

Limitations notwithstanding, findings hold pragmatic value; detailed understanding of the ‘problem’ represented in inconsistent adherence to guidelines and factors enabling comprehensive responding within busy public mental health services can support practice improvement. Findings indicate that although not sufficient, having and optimising use of necessary resources is a key to adherence to guidelines. Psychiatrists must be afforded time and be appropriately equipped. Careful consideration should be given at the service level to how expensive medical time can be used to best effect and psychiatrists should be afforded opportunity to develop familiarity with guidelines for prescribing antihypertensive/anti-lipidaemic agents and expertise needed to safely prescribe. More fundamentally, however, psychiatrists' ambivalence about responsibility and capacity to effect change in an apparently intractable problem must be addressed. Given the importance of professional norms in shaping observed practice, it is incumbent on diligent practitioners to actively encourage proactive attitudes in peers and trainees, and on all involved to build the service culture needed for change.

However, because mental health services and psychiatrists are but parts of a system of care, establishment of the consensus around responsibility of the diverse stakeholders and coordination of care sought by participants in this study and others^24^ must be a strategic priority. In this regard, consideration must be given to the broader sociopolitical context and the multiple pressures on healthcare. The efficiency imperative described by psychiatrists in this study as a key influence on clinical practice is a challenge faced internationally. As articulated by the president of the Royal College of Psychiatrists, Professor Sir Simon Wessely:

‘We are being asked to do more with less. We are campaigning and saying that people need to be more open about mental health problems and come forward earlier … but when they do, we find ourselves with less resources to treat them and they are getting short-changed.’^25^

Appropriate funding must accompany increasing expectations if healthcare and outcomes are to be improved in any sustainable way.

We conclude that psychiatrists have an opportunity to contribute to a reduction in cardiovascular disease that is prematurely killing people with SMI, but, to be effective, this must be part of strategically planned service- and system-wide responses. With collaboration central to ensuring practice improvement, the challenge for psychiatrists and mental health services is to overcome inertia and create the culture and partnerships that will support holistic care, which is critical to reducing the health inequities of people with SMI. As a first step, stakeholders must be engaged collectively in establishing consensus around roles, responsibilities and communication pathways. In relation to institutionalisation of evidence-based practice more generally in psychiatry, it seems that both cultural and structural change is needed to enable doctors to deliver the care to which they aspire. As argued by Grol & Grimshaw,^11^ ‘even where doctors are aware of the evidence and are willing to change, to alter well established patterns of care is difficult, especially if the clinical environment is not conducive to change.’
